# The crystal structure of AcrR from *Mycobacterium tuberculosis* reveals a one‐component transcriptional regulation mechanism

**DOI:** 10.1002/2211-5463.12710

**Published:** 2019-08-20

**Authors:** Sung‐Min Kang, Do‐Hee Kim, Chenglong Jin, Hee‐Chul Ahn, Bong‐Jin Lee

**Affiliations:** ^1^ The Research Institute of Pharmaceutical Sciences, College of Pharmacy Seoul National University Seoul Korea; ^2^ Department of Pharmacy Dongguk University‐Seoul Ilsandong‐gu Goyang Korea

**Keywords:** AcrR, *Mycobacterium tuberculosis*, transcriptional regulator, X‐ray crystallography

## Abstract

Transcriptional regulator proteins are closely involved in essential survival strategies in bacteria. AcrR is a one‐component allosteric repressor of the genes associated with lipid transport and antibiotic resistance. When fatty acid ligands bind to the C‐terminal ligand‐binding cavity of AcrR, a conformational change in the N‐terminal operator‐binding region of AcrR is triggered, which releases the repressed DNA and initiates transcription. This paper focuses on the structural transition mechanism of AcrR of *Mycobacterium tuberculosis* upon DNA and ligand binding. AcrR loses its structural integrity upon ligand‐mediated structural alteration and bends toward the promoter DNA in a more compact form, initiating a rotational motion. Our functional characterization of AcrR and description of the ligand‐ and DNA‐recognition mechanism may facilitate the discovery of new therapies for tuberculosis.

**Abbreviations**
EMSA: electrophoretic mobility shift assay
HTH: helix‐turn‐helix
PDB: Protein Data Bank
RMSD: root mean square deviation
SeMet: selenomethionine
TetR: tetracycline repressor

In bacteria, adaptive responses to changes in living conditions are essential survival strategies mediated by transcriptional regulator proteins. The tetracycline repressor (TetR) family member AcrR is a well‐characterized functional protein of the transcriptional regulation system that confers resistance to the antibiotic tetracycline 1, 2. AcrR has a strong affinity for DNA. It binds to the operator site and represses the transcription of its own gene. When AcrR binds a fatty acid or tetracycline, it loses its affinity for the operator 3, 4. This effector binding to the ligand‐binding domain of AcrR invokes an allosteric cascade, resulting in a conformational change in the DNA‐binding domains 5, 6. The homodimeric model of the *Mycobacterium tuberculosis* AcrR contains a DNA‐binding domain with a helix‐turn‐helix (HTH) motif and a ligand‐binding domain with a dimerization interface 7, 8.

AcrR is responsible for antibiotic resistance in a wide range of Gram‐negative bacteria. As a mechanistic analog, the binding of the tetracycline–magnesium complex to AcrR abolishes the DNA‐binding affinity of AcrR and allows transcription of the multidrug efflux complex AcrAB 8, 9. Then, AcrR is released from the target promoter DNA, and AcrAB is expressed. The expression of the AcrAB efflux complex protects the bacterial cell by exporting toxic substances such as antibiotics out of the cell 10, 11. Although earlier work in this field has already revealed that the recognition of divalent metal ions by AcrR might be related to transcriptional regulation mechanisms, the allosteric conformational changes upon metal binding remain enigmatic 12, 13.

However, *M*. *tuberculosis* has a mycobacterial cell wall, conferring resistance to antibiotics that inhibit cell wall biosynthesis. This inhibition of cell wall biosynthesis is associated with lipid transporters 14. AcrR from *M*. *tuberculosis* also participates in regulating these transporter proteins that export fatty acids to the cell wall. As AcrR regulators, fatty acids can also induce ligand‐mediated regulation of transcription, similar to tetracycline, triggering rotational motion of the entire protein 15, 16.

Here, we address the structural insight into the allosteric communication of AcrR based on limited proteolysis, CD spectroscopy, electrophoretic mobility shift assay (EMSA), X‐ray crystallography, and structural analysis. We focus on the structural mechanism of DNA or ligand binding depending on structural integrity. AcrR is an important model for allosteric gene regulation at the transcription level. Investigation of the ligand‐ or DNA‐recognition system of AcrR can engender improved biological understanding and may facilitate the discovery of new antibiotics 17, 18.

## Results and Discussion

### Overall structure of AcrR

**Figure 1:**
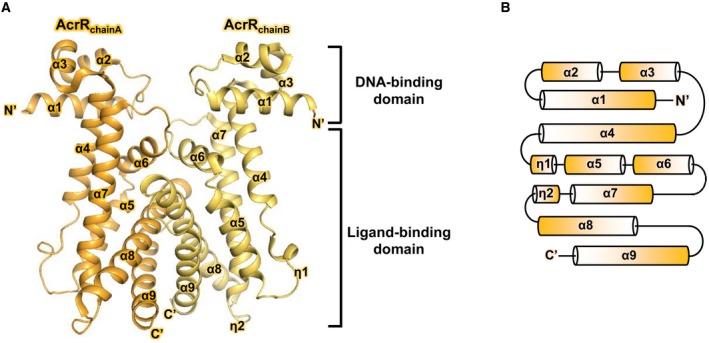
Overall structure of AcrR. (A) Ribbon representation of the AcrR homodimer. Chains A and B are shown in orange and yellow, respectively. The DNA‐ and ligand‐binding domains are indicated in the figure, respectively. (B) Schematic diagram showing secondary structure architecture of AcrR.

The asymmetric unit of the AcrR crystal structure contains a homodimeric assembly (Fig. 1A). *Mycobacterium tuberculosis* AcrR contains nine α‐helices and two 3_10_‐helices (η) in the following order: α1 (residues: 14–28), α2 (residues: 37–44), α3 (residues: 48–55), α4 (residues: 58–75), η1 (residues: 80–82), α5 (residues: 91–103), α6 (residues: 107–116), α7 (residues: 123–144), η2 (residues: 145–147), α8 (residues: 154–181), and α9 (residues: 188–207; Fig. 1B). Secondary structure was analyzed using the 2Struc server 19.

**Figure 2:**
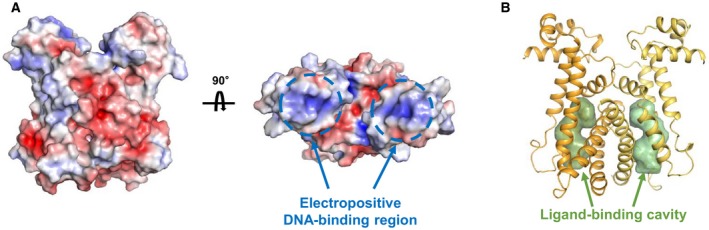
Electrostatic surfaces of AcrR and ligand‐binding cavity. (A) Electrostatic surface potential of AcrR with front and 90˚ horizontally rotated view. Each electropositive DNA‐binding region (α2–α3) of AcrR is indicated by dotted lines. (B) The location of the ligand‐binding cavity in AcrR. Ligand‐binding cavities are indicated by green arrows.

AcrR is organized into two functional units: the N‐terminal DNA‐binding domain and the C‐terminal ligand‐binding domain. The N‐terminal DNA‐binding domain includes helices α1, α2, and α3. Helices α2 and α3 form the HTH motif. The positively charged surface of helices α2 and α3 can recognize the DNA major groove, constituting an interface that binds the negatively charged phosphate backbone of DNA (Fig. 2A). The electrostatic potential surface was calculated using the Adaptive Poisson‐Boltzmann Solver method 20. The C‐terminal ligand‐binding domain is composed of helices α4–α9. According to the results from KVFinder 21, there is a large ligand‐binding pocket with a cavity volume of 543–560 Å^3^ between helices α4–α7 (Fig. 2B). Helices α6, α8, and α9 form the dimerization interface.

**Figure 3:**
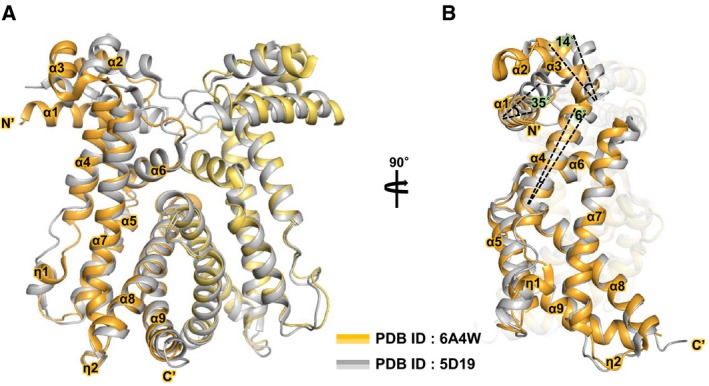
Structural comparison of 6A4W and 5D19 of the AcrR regulator. (A) Superimposition of the dimeric structures of 6A4W and 5D19 (yellow, 6A4W; black, 5D19). Helices of both structures are also numbered. The arrow indicates a change in view compared with that in (B). (B) Side view of superimposition. This view depicts a rigid body rotational motion of the two structures. Comparative statistical values have been marked in this figure.

Additionally, structural comparison between two AcrRs from *M. tuberculosis*, Protein Data Bank (PDB) code 6A4W and 5D19, was conducted to further obtain structural information on *M. tuberculosis* AcrR 22. Interestingly, two conformations of AcrR with different space groups, *P*6_1_ (6A4W) and *P*2_1_2_1_2_1_ (5D19), have been observed. Two structures exhibit dimeric arrangement in crystallographic symmetry, and each subunit of AcrR consists of nine α‐helices (α1–α9). In both structures, α5, α6, and α7 helices were folded to create a ligand‐binding cavity, and α8 and α9 form the dimerization interface. However, considerable structural deviations in the N‐terminal DNA‐binding domain (α1–α3) originate from the α4 helix. Superimposition of the dimeric structures of 6A4W and 5D19 results in an overall root mean square deviation (RMSD) of 2.0 Å (Fig. 3A). The difference between the two conformations originates from 6˚ rotational motion in the α4 helix of the 6A4W with respect to the 5D19 which results in rigid body rotations in the α1 helix (35°) and α3 helix (14°; Fig. 3B). Based on this structural comparison, it can be inferred that ligand binding triggers a rotational motion within the regulator protein. This movement seems to prohibit the binding of DNA to the regulator protein AcrR.

In a normal state, the expression of the AcrAB complex is repressed by AcrR via tight binding. However, the C‐terminal tunnel‐like ligand‐binding cavity of AcrR can accommodate various ligands, such as tetracycline, Mg^2+^, or palmitate. Once this cavity is occupied by those ligands, a conformational change in the DNA‐binding domain is triggered by an allosteric cascade, interfering with the repression of AcrAB 2, 23, 24. The protein exports antibiotics outside the cell, which contributes to drug resistance, and maintains bacterial pathogenicity by regulating the transport of cell wall lipids by lipid transporters 22, 25. Thus, solving the interaction mechanism of this one‐component allosteric gene regulation system should illuminate the drug resistance mechanism of *M. tuberculosis* 26, 27, 28.

### Transcriptional regulation analogy of AcrR

**Figure 4:**
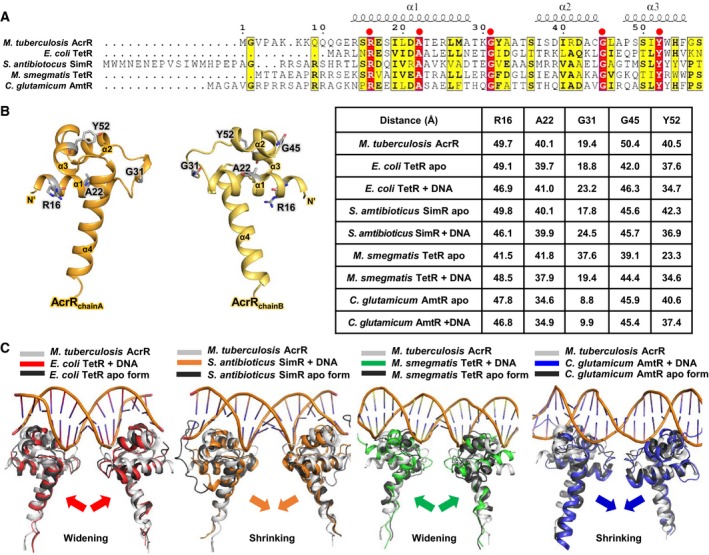
Sequence and structural alignment of *Mycobacterium tuberculosis* AcrR, *Escherichia coli* TetR, *Streptomyces antibioticus* SimR, *Mycobacterium smegmatis* TetR, and *Corynebacterium glutamicum* AmtR. (A) Sequence alignment of the N‐terminal regions of the proteins, including helices α2–α3 in the HTH DNA‐binding domain. The secondary structural elements of *M. tuberculosis* AcrR are shown above the alignment. Identical and similar residues are highlighted in red and yellow, respectively. (B) Left: the locations of highly conserved residues (R16, A22, G31, G45, and Y52) in AcrR. Right: the distances between highly conserved residues between each dimerized chain of AcrR and of the apo form and DNA‐bound form of its structural homologs. (C) Structural superposition of *M. tuberculosis* AcrR (gray) and each DNA‐bound structure and apo structure (black) listed above as follows: *E. coli* TetR (red); *S. antibioticus* SimR (orange); *M. smegmatis* TetR (green); and *C. glutamicum* AmtR (blue). Narrowing and widening upon DNA binding are also indicated by arrows in each corresponding color.

To investigate the DNA‐binding mode of TetR‐type transcriptional regulators, sequence alignment was performed using Clustal Omega 29 with four currently reported DNA‐bound TetR‐type transcriptional regulators and visualized using espript 3.0 30 (Fig. 4A). The sequence alignment result of the N‐terminal region of AcrR, including the HTH DNA‐binding domain of helices α2 and α3, shows a highly conserved tyrosine residue in the α3 helix and glycine residues in each loop between α1 and α2 and between α2 and α3, corresponding to a hydrogen‐bonded turn. Among these conserved residues, the tyrosine residue contributes to maintaining the proper function of the DNA‐binding domain and the structural integrity of the protein, and glycine residues form the hydrogen‐bonding‐mediated turn in the DNA‐binding conformation of the HTH domain 31, 32, 33. Other highly conserved arginine and alanine residues in α1 contribute to base‐specific interactions with DNA. In particular, the electropositive charge of the arginine residue contributes to the appropriate positioning of AcrR on the negatively charged phosphate backbone of DNA. Arginine might be critically involved in the recognition of double‐stranded DNA 2, 6, 23, 34. The locations of five highly conserved residues in the N‐terminal structure of AcrR are illustrated in Fig. 4B.

Additionally, for a detailed comparison, the structural similarity of AcrR and four DNA‐bound structures was analyzed using the Dali server 35. These structural homologs included (a) TetR from *Escherichia coli* 36; (b) SimR from *Streptomyces antibioticus* 6; (c) TetR from *Mycobacterium smegmatis* 37; and (d) AmtR from *Corynebacterium glutamicum* 34 (Table 1). Analysis using the Dali server was also conducted to determine the apo form of each DNA‐bound homolog. These apo forms include (a) TetR from *E. coli* 38; (b) SimR from *S. antibioticus* 23; (c) TetR from *M. smegmatis* 37; and (d) AmtR from *C. glutamicum* 34 (Table 2). Generally, the structural homologs show very similar statistical values (RMSDs of 3.0–4.7 and *Z*‐scores of 8.5–13.7) despite their low sequence similarity. The distances of five highly conserved residues (Arg16, Ala22, Gly31, Gly45, and Tyr52) in Fig. 4A between each pair of dimerized chains are also described in Fig. 4B.

**Table 1:** Structural similarity comparison of AcrR with DNA‐bound structures using Dali sever. RMSD, root mean square deviation.

Protein name	Source	PDB code (used chain)	*Z*‐score	RMSD (Å)	Number of aligned Cα	Sequence identity (%)
TetR	*Escherichia coli*	1QPI (A)	10.4	3.7	164	15
SimR	*Streptomyces antibioticus*	3ZQL (A, B, C, D)	10.2–10.6	3.5–3.8	161–165	14
TetR	*Mycobacterium smegmatis*	4JL3 (A, B, C, D)	12.5–13.7	3.0	166–170	16–20
AmtR	*Corynebacterium glutamicum*	5DY0 (A, B, C, D)	8.5–9.4	4.0–4.5	121–148	19–23

**Table 2:** Structural similarity comparison of AcrR with apo structures using Dali sever. RMSD, root mean square deviation.

Protein name	Source	PDB code (used chain)	*Z*‐score	RMSD (Å)	Number of aligned Cα	Sequence identity (%)
TetR	*Escherichia coli*	2TCT (A)	10.6	3.9	167	14
SimR	*Streptomyces antibioticus*	2Y2Z (A)	9.7	3.5	161	15
TetR	*Mycobacterium smegmatis*	4JKZ (A)	10.4	4.7	125	24
AmtR	*Corynebacterium glutamicum*	5DXZ (A)	9.5	4.2	152	23

Because of similarities between the DNA‐bound structures and apo AcrR, we used the reported structures as a template to model the putative AcrR–DNA complex. The dimeric structure of AcrR was superimposed with respect to the HTH domain onto each DNA‐bound structure (Fig. 4C). In this DNA‐binding model of AcrR, the N‐terminal DNA‐binding domain is composed of helices α1–α3 (residues 14–55). Helix α2 (residues 37–44) and the recognition helix α3 (residues 48–55) form the HTH motif, which packs against helix α1 for stabilization. In the HTH motif, both α2 and α3 are very rich in positively charged surfaces. Upon binding to DNA, this N‐terminal domain is bent toward the DNA. The recognition helix α3 is inserted into the turns of the DNA major groove, and helix α2 supports the DNA binding. It will be interesting to further examine the operator recognition mechanism of AcrR in association with other TetR family proteins 6, 34, 36, 37, 39.

The distances between each Cα from the 4th conserved glycine in helices α2 and α3 of each monomer were compared. The distance in *M. tuberculosis* AcrR was ~ 10–20% longer (50.4 Å) than the corresponding distances in *E. coli* TetR (46.3 Å for the DNA‐bound form, 42.0 Å for the apo form), *S. antibioticus* SimR (45.7 Å for the DNA‐bound form, 45.6 Å for the apo form), *M. smegmatis* TetR (44.4 Å for the DNA‐bound form, 39.1 Å for the apo form), and *C. glutamicum* AmtR (45.4 Å for the DNA‐bound form, 45.9 Å for the apo form). Recent structural and thermodynamic studies of protein–DNA complexes show that not only the DNA but also the protein undergoes conformational changes to facilitate favorable interactions with DNA 40, 41. This theory is referred to as the ‘induced‐fit mechanism’. The long distance between the 4th conserved glycine between helices α2 and α3 could present a spatial challenge for DNA recognition compared to the shorter distances in other homologous proteins. This difference could be a crucial reason for the failure to obtain DNA‐bound crystals *in vitro*. In addition, during DNA binding, *S. antibioticus* SimR and *C. glutamicum* AmtR, which do not show a large distance between the 4th conserved glycine positions, have a smaller distance in the DNA‐bound conformations than in their apo forms, but *E. coli* TetR and *M. smegmatis* TetR form have wider DNA‐binding conformations, showing a considerable difference in distance between the 4th conserved glycine. It can be inferred that the N‐terminal DNA‐binding domain of AcrR undergoes widening and shrinking in the absence and presence of DNA. This structural alteration is predicted to facilitate or hinder the protein–DNA interaction 42, 43.

### On–off interaction of AcrR–DNA upon ligand binding

**Figure 5:**
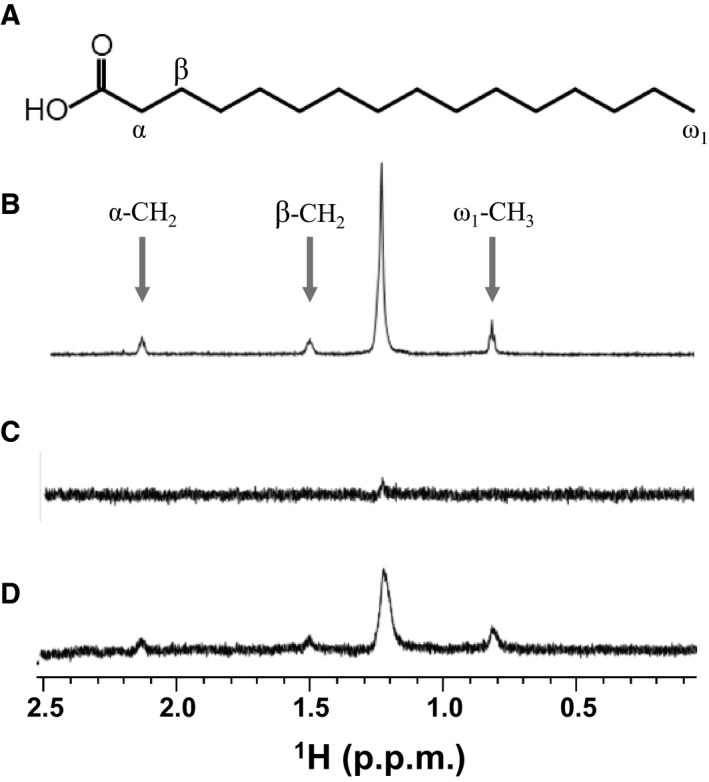
STD analysis of palmitate. (A) Structure of palmitate. (B) Reference ^1^H spectrum of palmitate. (C) STD‐NMR spectrum of palmitate in the absence of AcrR. (D) STD‐NMR spectrum of palmitate in the presence of AcrR. The residues and peaks corresponding to the α‐ and β‐methylene groups and ω1 methyl group are indicated in the structure and spectra, respectively. The residual methylene groups positioned about 1.2–1.3 p.p.m. are not indicated.

**Figure 6:**
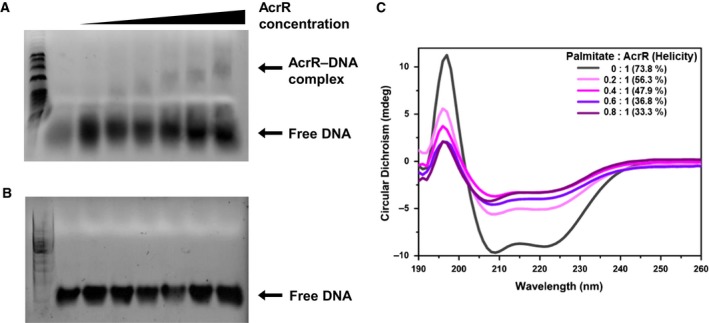
DNA‐binding properties and structural folding in the absence and presence of ligand palmitate. (A) EMSA experiment testing the binding of AcrR to its own promoter DNA in the absence of palmitate. (B) EMSA experiment testing the binding of AcrR to its own promoter DNA in the presence of 0.1 mm palmitate. In both assays, 0.01 mm promoter DNA was incubated (20 min at 4 °C) with (+) or without (−) 0 mm to 1 mm AcrR. The formation of DNA–protein complexes was observed only in the absence of palmitate as the ratio of protein to DNA was increased. As DNA binds to a large amount of protein, the bands corresponding to the DNA–protein complex move upward. (C) An overlay of the CD spectra of AcrR (black) with increasing proportions of palmitate (thickening purple). The ratios and helicities corresponding to each line are indicated in the upper right area of the graph.

Since it has been suggested that palmitate might be a natural ligand of the TetR‐type transcriptional regulator 44, 45, the binding of AcrR with palmitate was monitored in a saturation transfer difference (STD)‐NMR experiment. First, a reference 1D ^1^H NMR spectrum of the palmitate was obtained, and the analysis of ^1^H peaks showed that the α‐ and β‐methylene groups, the ω_1_‐methyl group, and the rest of the methylene groups overlapped at ~ 1.2–1.3 p.p.m. (Fig. 5A,B). To confirm the perturbation of palmitate by the selective on‐resonance irradiation, an STD experiment with palmitate in the absence of the AcrR was conducted, which showed the absence of STD signals from direct irradiation of palmitate (Fig. 5C). An STD spectrum of palmitate in the presence of AcrR showed methyl and methylene proton signals of palmitate, which reveals the binding between AcrR and palmitate (Fig. 5D). An EMSA and CD spectroscopy were conducted to elucidate the ligand‐mediated conformational change in AcrR. In the EMSA experiment, the promoter DNA concentration was maintained at 0.01 mm, and the concentration of protein was increased from 0 to 1 mm. As the amount of DNA bound to protein increased, the bands corresponding to the DNA–protein complex were gradually generated and shifted upward (Fig. 6A). The smearing and upward movement of the AcrR–DNA complex band in the EMSA results is discussed below. At first, the interaction with weak binding affinity exhibited a smearing band shift, and discrete bands were not seen. This is typical in EMSA when DNA has weak‐to‐moderate affinity with the target protein 46, 47. Based on this, it is likely that the binding mode between AcrR and DNA shows fast exchange on EMSA, which is typically observed when ligands bind with a low‐to‐moderate affinity. AcrR–DNA might also bind to more than one binding site, resulting in the formation of multimeric complexes or aggregates 48, 49.

An additional experiment was conducted to monitor the effect of palmitate on AcrR–DNA complex formation. When 0.1 mm palmitate was present in the AcrR–DNA mixture, no shifted AcrR–DNA bands were observed (Fig. 6B). Although 0.1 mm palmitate is a small amount relative to 1 mm AcrR, considering the weak binding affinity of the AcrR–DNA complex shown in Fig. 6A, it would be sufficient to inhibit the interaction between AcrR and DNA.

To confirm the structural transition that occurs during the binding of palmitate to AcrR, we compared the CD spectra of AcrR in the absence of palmitate and in the presence of an increasing proportion of palmitate (0–80% of the AcrR concentration). These spectra are overlaid as shown in Fig. 6C. Upon titration with palmitate, the CD spectrum of AcrR showed decreased α‐helicity, indicating that AcrR loses a considerable amount of structural integrity upon ligand binding, although the overall architecture remains largely α‐helical 50, 51. The α‐helicity values of the protein were calculated by CDNN software 52. According to the CDNN calculation algorithm, the magnitude of mdeg can be used to calculate the α‐helicity at an equal concentration of protein 53.

The results of the titration experiment are consistent with those of the EMSA experiment. In accordance with the decreased structural integrity observed from the CD spectra during titration, the EMSA study shows the decreased binding of DNA and AcrR. Subsequently, the transcription of drug resistance‐ and pathogenesis‐associated genes is initiated by DNA released from repressor proteins. Since tuberculosis is a serious disease, and numerous patients worldwide are infected with drug‐resistant strains, understanding the on–off transcriptional regulatory mechanism of the TetR type will be helpful in increasing the efficiency of existing drugs 54, 55, 56.

### Structural integrity of AcrR upon cofactor binding

To obtain insight into the structural integrity of AcrR, limited proteolysis of AcrR using trypsin was performed with DNA and the potential cofactors palmitate, Mg^2+^, and tetracycline. We examined the effect of the binding of promoter DNA to AcrR. The results of the initial short‐timescale (5 min) and long‐timescale (10–20 min) proteolysis experiments showed that the addition of palmitate facilitates the proteolysis of both AcrR alone and the AcrR–DNA complex. In the presence of palmitate, AcrR showed more degradation upon the addition of protease, as evidenced by a weaker magnitude of the stained band at the protein mass than that of AcrR with only pepsin added. However, the other putative cofactors, tetracycline and magnesium, did not noticeably affect the proteolysis of AcrR or yielded only a negligibly increased degradation pattern compared to that of AcrR with no cofactor upon exposure to trypsin.

According to the literature regarding homologous proteins, tetracycline and magnesium also affect the conformational changes of the AcrR protein in *E. coli* 5, 17, 36, 57. However, in the conformational change mechanism, tetracycline and magnesium seem to have less of a tendency to undermine structural integrity than palmitate. Based on our results, it can be inferred that tetracycline and magnesium contribute to the conformational change of the AcrR protein through mechanisms different from that of palmitate.

**Figure 7:**
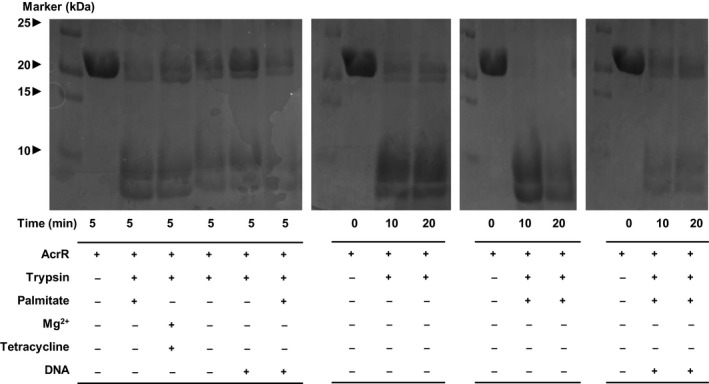
Limited proteolysis of AcrR. AcrR treated with trypsin in the absence and presence of DNA and various cofactors visualized by SDS/PAGE. The reagents added to each well are indicated in the table below. First panel: proteolyzed yields after 5 min of proteolysis. The negative control contains only AcrR, and the positive control contains AcrR and trypsin only. Cofactors and DNA contribute to proteolytic degradation in an antagonistic manner. Second panel: trypsin‐proteolyzed yields of AcrR every 10 min. Third panel: trypsin‐proteolyzed yields of AcrR and palmitate every 10 min. Fourth panel: trypsin‐proteolyzed yields of AcrR–DNA complex and palmitate every 10 min.

In contrast, the addition of promoter DNA substantially decreased the rate of AcrR proteolysis, showing a more preserved band than that of DNA‐free AcrR, indicating that the AcrR–DNA interaction renders AcrR more resistant to protease and structurally better organized. However, even in the presence of DNA, ligand binding to AcrR made AcrR susceptible to proteolytic cleavage (Fig. 7).

These results suggest that partially unfolded AcrR might undergo a structural transition from a random‐coil or near‐helix state to a more helical, fully structured state upon interaction with DNA. In contrast, analysis of the α‐helical CD signals of AcrR in the presence of palmitate reveals that the structural integrity of AcrR is disrupted via the AcrR–ligand interaction. AcrR transitions from a structurally well‐organized and rigid state to a conformationally flexible state upon ligand binding. According to recent studies, ligand binding to these types of transcriptional regulators triggers global reorganization of protein at the DNA‐binding domain, leading to the widening of the DNA‐binding domain and resulting in the release of DNA 55, 58. Furthermore, palmitate has been demonstrated to be a possible ligand of regulatory proteins for protein binding affinity 22. Based on the limited proteolysis results, it can be inferred that decreased structural integrity due to the effect of palmitate led to conformational changes. Our results suggest that in the presence of the cofactor palmitate, AcrR is prone to proteolysis and loses its structural integrity relative to cofactor‐free wild‐type AcrR 6, 57, 59.

## Conclusion

In this study, we solved the crystal structure of AcrR, advancing the current understanding of one‐component transcriptional regulatory mechanisms. Our structural analysis revealed the structural transition of helices α2–α3 in the HTH DNA‐binding motif, which bend toward the promoter DNA in a more compact conformation. Furthermore, we postulated the structural alteration of AcrR after complexation with a ligand. The ligand‐mediated conformational change in AcrR, especially in the DNA‐recognition domain, releases the promoter DNA repressed by AcrR, which in turn initiates the expression of genes associated with lipid transporters and antibiotic resistance. This conformational change induces rotational motion of the AcrR structure, which results in a loss of structural integrity. Based on a comprehensive study of the molecular mechanism and functional characterization of AcrR, this article provides evidence that compounds capable of inhibiting AcrR could improve the therapeutic index of current tuberculosis drugs.

## Materials and methods

### Cloning and purification

The *AcrR* gene was amplified by PCR using the following primers: forward, 5′‐GGAATTCCATATGGAGAGGTCACGAGAATCG‐3′; and reverse, 5′‐CCGAAGCTTTTATGTCTCCTCCAGGAGGAC‐3′. The PCR product and pET28b vector were double‐cleaved by *Nde1* and *Hind3* and ligated resulting in an N‐terminal (MGSSHHHHHHSSGLVPRGSH) tag. For crystallization, the cloned plasmids were transformed into *E. coli* Rosetta2 (DE3) pLyss competent cells (Novagen, Madison, WI, USA). The cells were grown at 37 °C in LB until the OD_600_ reached 0.6. Protein overexpression was induced by the addition of 0.5 mm isopropyl IPTG, and additional incubation was conducted at 37 °C for 4 h. The cultured cells were harvested by centrifugation at 11 355 ***g*** and 4 °C, suspended in buffer A (20 mm Tris/HCl, pH 7.9, and 500 mm NaCl) with 5% glycerol by volume, and lysed by ultrasonication. After centrifugation for 1 h at 28 306 ***g***, the supernatant containing soluble proteins was purified using similar procedure with previous paper 47. Final sample was concentrated to 15 mg·mL^−1^, and the purity of the protein was verified by SDS/PAGE. Selenomethionine (SeMet)‐labeled protein was obtained by the same procedure, except that cells containing the SeMet‐labeled protein were grown in M9 medium containing additional essential amino acids.

### Crystallization, data collection, and processing

Initial crystal screening of the purified AcrR was performed using Wizard Kits (Rigaku Reagents, Bainbridge Island, WA, USA) by mixing 1 μL of protein solution at 15 mg·mL^−1^ in 20 mm Tris, pH 7.5, and 150 mm NaCl with 1 μL of reservoir solution. Crystals were grown in the crystallization solution of 100 mm MES, pH 6.0, and 1.26 m ammonium sulfate using the sitting‐drop vapor diffusion method at 20 °C. The crystallization solution with 20% glycerol was used as cryoprotectant. The crystals were flash‐cooled in liquid nitrogen prior to data collection. The data collection was conducted using an ADSC Quantum Q270r CCD detector at beamline 5C of the Pohang Light Source, Republic of Korea. The AcrR crystals belonged to the *hexagonal* space group *P*6_1_ with unit cell parameters of *a* = 118.752 Å, *b* = 118.752 Å, and *c* = 93.456 Å for the SeMet‐labeled crystal and *a* = 118.154 Å, *b* = 118.154 Å, and *c* = 90.906 Å for the native crystal. All raw data were scaled and processed by HKL2000 60. A set of SAD data at 2.80 Å resolution from a SeMet‐labeled crystal was used to solve the phase problem and refined into 6A4L. 6A4W was solved by the molecular replacement method employing the refined model of 6A4L using 2.60 Å data of the native crystal. 6A4W was used for structural analysis in this paper. Detailed statistical information on the structures is shown in Table 3. phenix 61 was first used to automatically build the model, and coot 62 was utilized to provide the starting model for refinement. The *R*_work_/*R*_free_ values 63 of the SeMet and the native final models obtained using REFMAC and phenix 61, 64 were 21.3/25.9% and 20.0/24.9%, respectively. The overall geometry validation was conducted using molprobity 65, and the results showed that 96.39% of the residues were in the favored region of the Ramachandran plot, and an additional 3.09% were in the allowed region in the native structure. All figures were generated using pymol (The PyMOL Molecular Graphics System, Version 1.3 Schrödinger, LLC., Cambridge, MA, USA).

**Table 3:** Data collection and refinement statistics for SeMet and native structures.

Data set	SeMet	Native
(a) Data collection details. Values in parentheses are for the highest‐resolution shell		
X‐ray source	5C beamline of PLS, Korea	5C beamline of PLS, Korea
X‐ray wavelength (Å)	0.9793	0.9796
Space group	*P*6_1_	*P*6_1_
Unit cell parameters		
*a*, *b*, *c* (Å)	118.752, 118.752, 93.456	118.154, 118.154, 90.906
α, β, γ (°)	90.0, 90.0, 120.0	90.0, 90.0, 120.0
Resolution range (Å)	50.0–2.80	50.0–2.60
Molecules per ASU	AcrR homodimer	AcrR homodimer
Observed reflections (> 1σ)	581 657	123 109
Unique reflections	18 576	21 205
<*I*/σ(*I*)>	45.3 (6.70)^e^	28.7 (4.81)^e^
Completeness (%)	100.0 (100.0)^e^	94.3 (96.8)^e^
Multiplicity^a^	31.3 (31.7)^e^	5.9 (6.4)^e^
*R*_merge_ (%)^b^	9.6 (65.6)^e^	11.5 (67.6)^e^
CC_1/2_, CC	(0.969, 0.992)^e^	(0.878, 0.967)^e^
(b) Refinement statistics		
*R*_work_ c (%)	21.3	20.0
*R*_free_ d (%)	25.9	24.9
No. of atoms/average *B* factor (Å^2^)	3211/43.0	3186/55.0
RMSD^f^ from ideal geometry		
Bond distance (Å)	0.006	0.006
Bond angle (°)	1.344	1.331
Ramachandran statistics		
Most favored regions (%)	92.31	96.39
Additional allowed regions (%)	6.92	3.09
PDB accession code	6A4L	6A4W

a *N*_obs_/*N*_unique_.

b *R*_merge_ = Σ (*I −* 〈*I*)/Σ〈*I*.

c *R*_work_ = Σ*_hkl_*||*F*_obs_| − *k*|*F*_calc_||/Σ*_hkl_*|*F*_obs_|.

d *R*_free_ was calculated in the same manner as *R*_work_ but with 5% of the reflections excluded from the refinement.

e Values in parentheses indicate the highest‐resolution shell.

f RMSD was calculated using REFMAC.

### STD‐NMR experiment

NMR experiments were conducted at 298 K using an AVANCE 800 MHz spectrometer equipped with a cryogenic probe (Bruker BioSpin, Billerica, MA, USA), and topspin 3.5 software (Bruker BioSpin) and iNMR (http://www.inmr.net) were utilized for data processing and visualization. The NMR sample was prepared in a buffer containing 20 mm MES, pH 6, and 50 mm NaCl, 10% D_2_0, and 5% DMSO. A ^1^H NMR spectrum of 30 μm palmitate was recorded as a reference spectrum. To identify the binding of palmitate with AcrR, STD‐NMR spectra were recorded in the absence and the presence of 1 μm AcrR using the pseudo‐2D pulse sequence, stddiff. On‐ and off‐resonance irradiations were applied at chemical shifts of 7.5 and −30 p.p.m., respectively.

### EMSA

Electrophoretic mobility shift assay was conducted to distinguish the binding affinity of AcrR for promoter DNA in the presence or absence of palmitate. Palmitate was dissolved completely in DMSO to make a stock solution. A 24‐base pair DNA fragment in a palindromic form from the upstream region (promoter DNA) of AcrR was added to the proteins. The palindromic sequence was as follows: forward, TTTCTTGGCGGGAACGCCCACTGG; and reverse, CCAGTGGGCGTTCCCGCCAAGAAA. The dsDNA and proteins were prepared in buffer (20 mm Tris, pH 7.5, and 150 mm NaCl). Varying amounts of AcrR protein were mixed with DNA and palmitate in a final volume of 10 μL and incubated for 20 min at 4 °C. The total binding solutions were loaded onto 0.8% agarose gels in 0.5 × TBE (45 mm Tris/borate, 1 mm EDTA) buffer, and the results were visualized using a Gel Doc (Bio‐Rad, Hercules, CA, USA).

### CD spectroscopy

The CD measurements of AcrR and palmitate‐added AcrR were conducted in a Chirascan‐plus spectropolarimeter (Applied Photophysics, Leatherhead, UK) at 20 °C using a 1 mm light path cell. All experiments were performed in buffer (20 mm Tris, pH 7.5, and 150 mm NaCl) at a protein concentration of 25 μm. Palmitate titration was conducted five times to measure the CD spectra, and the palmitate concentration varied from 0 to 20 μm (a maximum of 80% of the protein concentration). CD scans were taken from 260 to 190 nm with a 1 nm bandwidth and a scan speed of 100 nm·min^−1^. Three scans were averaged, and the solvent signal was subtracted.

### Limited proteolysis

To focus on structural integrity upon promoter DNA and ligand binding, limited proteolysis of the AcrR using bovine trypsin (Sigma‐Aldrich, St. Louis, MO, USA) was performed 66. AcrR (100 μm) was incubated with trypsin at a mass ratio of 1000 : 1 in buffer (20 mm Tris, pH 7.5, and 150 mm NaCl) at 4 °C with DNA (100 μm), palmitate (100 μm), MgCl_2_ (10 mm), and tetracycline (10 mm). The same promoter DNA was used as that in the EMSA experiment. After 1, 5, and 10 min of incubation, samples were taken, and the reactions were stopped by adding SDS/PAGE loading buffer, boiled, and examined by SDS/PAGE.

## Conflict of interest

The authors declare no conflict of interest.

## Author contributions

SMK and DHK conceived the study, designed the experiments, analyzed the data, and wrote the manuscript. SMK, DHK, and CJ performed the experiments.. SMK, DHK, HCA, and BJL edited the manuscript.

## Data Availability

The structures have been deposited in the PDB under accession codes 6A4L and 6A4W for the model from the SeMet‐labeled crystal and for the model from the native crystal, respectively.
